# Care Coordination and Optimization in Geriatric Surgery (COGS): A Pilot Program to Improve Surgical Outcomes in Older Adults

**DOI:** 10.1111/jgs.70474

**Published:** 2026-05-23

**Authors:** Gabrielle A. Scannell, Carole A. Baraldi, Randall Rupper, Michelle T. Mueller, Rachel J. Brenner, Thomas White, Brian C. Sauer, Jared Lareno Hansen, G. Paul Eleazer

**Affiliations:** ^1^ Division of Geriatrics, Department of Internal Medicine University of Utah Spencer Fox, Eccles School of Medicine Salt Lake City Utah USA; ^2^ Geriatrics Research, Education, and Clinical Center (GRECC), Department of Veterans Affairs George E. Wahlen VAMC Salt Lake City Utah USA; ^3^ Vascular Surgery, Department of Veterans Affairs George E. Wahlen VAMC Salt Lake City Utah USA; ^4^ Northwest Permanente Medical Group Portland Oregon USA; ^5^ Division of Epidemiology, Department of Internal Medicine University of Utah Spencer Fox, Eccles School of Medicine Salt Lake City Utah USA; ^6^ Department of Veterans Affairs Salt Lake City Health Care System, IDEAS Center Salt Lake City Utah USA; ^7^ Department of Veterans Affairs Elizabeth Dole Center of Excellence for Veteran and Caregiver Research San Antonio Texas USA

**Keywords:** care coordination, frailty, geriatric surgery, interdisciplinary care, preoperative optimization

## Abstract

Older adults undergoing surgery are at increased risk for complications, functional decline, and prolonged recovery due to frailty and other age‐related vulnerabilities. To address these challenges, we developed the Care Coordination and Optimization in Geriatric Surgery (COGS) program, a pilot initiative integrating comprehensive geriatric assessment and targeted optimization into routine surgical care for Veterans. Using the Risk Analysis Index‐Clinical (RAI‐C) to identify higher‐risk patients, the COGS team—comprising geriatric providers, nursing, and surgical staff—provides individualized evaluation and interventions focused on nutrition, mobility, medication safety, cognition, and social support. By embedding geriatric expertise directly into the surgical clinic, the program fosters collaboration among surgeons and geriatricians, improving communication and supporting shared decision‐making about surgical readiness. Early experience demonstrates that COGS is both feasible and well‐received, enhancing the coordination of care for older Veterans and aligning surgical decisions with patients' goals and overall health status. Expansion of the program to additional surgical specialties and evaluation of short and long‐term outcomes are underway. The COGS model demonstrates how integrating geriatrics principles within surgical care can promote safer, more goal‐aligned surgery for older adults.

## Summary

- Key points
  ○ Frailty‐Informed Surgical Optimization: The COGS program systematically identifies high‐risk Veterans to ensure frailty, comorbidities, and functional status are incorporated into surgical planning.
  ○ Embedded Interdisciplinary Model: Embedding geriatricians and a geriatrics nurse to complete geriatric assessments directly into surgical clinics enables real‐time collaboration, shared decision‐making, and individualized, goal‐aligned care.
- Why does this paper matter?
  ○ This paper demonstrates how embedding geriatricians and a geriatrics nurse within surgical clinics can transform perioperative care for older adults. The COGS model offers a unique and potentially scalable framework for integrating frailty assessment and optimization into surgical workflows with the goals of improving outcomes and supporting broader implementation of age‐friendly, value‐based surgical care.

## Graphical abstract

The Care Coordination and Optimization in Geriatric Surgery (COGS) program is an initiative that targets geriatric assessment and multidisciplinary optimization to identify frailty and guide surgical decision‐making with the goal to improve perioperative care for older adults.

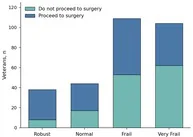

## 1 Introduction

Surgical care in older adults, particularly among Veterans, presents unique challenges due to age‐related physiological changes, multimorbidity, frailty, and cognitive impairment. One‐year postoperative mortality rates in older adults have been reported as high as 13.4%, with frailty emerging as a major risk factor [1, 2, 3]. Frailty, defined as decreased physiological reserve and increased vulnerability to stressors, is independently associated with adverse surgical outcomes, including postoperative complications, prolonged hospitalization, and increased mortality [4, 5, 6, 7, 8, 9, 10].

Preoperative geriatric assessment and optimization have been demonstrated to improve outcomes, reduce complications, and shorten length of stay [11, 12]. Emerging evidence suggests these approaches may also reduce postoperative delirium, preserve functional status, enhance surgical decision‐making, improve goal‐concordant care, and support adherence to multidisciplinary perioperative guidelines [13, 14, 15, 16, 17, 18, 19, 20, 21]. Despite growing recognition of the importance of geriatrics‐informed surgical care, we have been unable to identify any published models that directly involve geriatric providers and nurses in surgical clinics [22, 23].

Several well‐established perioperative geriatrics models have informed this work. The Duke Perioperative Optimization of Senior Health (POSH) program, originally described by McDonald et al. [24], integrates geriatric assessment into surgical care through a centralized clinic model and has demonstrated associations with improved postoperative outcomes in high‐risk older adults. Subsequent work from the Durham Veterans Affairs (VA) Medical Center further described structured approaches to delaying elective surgery to allow for optimization when appropriate [25]. In parallel, the American College of Surgeons (ACS) Geriatric Surgery Verification (GSV) Program has established standards to improve surgical care for older adults across institutions [26].

During the conceptual development of the Care Coordination and Optimization in Geriatric Surgery (COGS) program and prior to its implementation, we deliberately sought to learn from established perioperative geriatrics programs across the country. This included close engagement with the Duke POSH program, whose team graciously invited us to participate in an in‐person mini‐fellowship, as well as active collaboration with VA preoperative geriatrics programs in Denver, Puget Sound, and Gainesville. In addition, we participated in the VA Surgical Pause Practice, a national collaborative that uses frailty to identify and assess high‐risk surgical patients. These experiences directly informed the design and implementation of COGS.

Our program shares conceptual similarities with the Duke POSH program and other perioperative geriatric initiatives within the VA and ACS/GSV, particularly in its emphasis on preoperative risk stratification and multidisciplinary optimization of older adults. However, there are several important distinctions in structure, implementation, and clinical workflow.

Unlike Duke's POSH program, which is centered on a geriatrician‐led interdisciplinary preoperative clinic to which patients are referred, COGS is embedded directly within surgical clinics. The geriatrics team evaluates patients in real time alongside the operating surgeon and surgical staff during routine surgical visits. The COGS model facilitates direct, bidirectional communication at the point of care, allowing geriatric assessment findings and recommendations to be immediately incorporated into surgical decision‐making. In contrast to the Duke POSH program and similar clinic‐based models, the operating surgeon may not directly participate in the geriatric assessment encounter.

The COGS program is also intentionally designed to be scalable without requiring universal geriatric consultation or a standalone geriatric clinic. Our program relies on standardized screening tools, protocol‐driven interventions, and integration into existing perioperative workflows, allowing implementation across diverse practice environments with limited geriatrics workforce capacity.

While other programs helped us to provide critical frameworks for perioperative optimization within our own program, we were constrained from re‐creating them at our institution given space, funding, and staffing limitations. We needed an adaptable, flexible program that could be modified rapidly to fit the needs and limitations of our institution. Ultimately, we integrated our program into existing surgical clinic space and embedded a geriatrician and geriatrics nurse directly into surgical clinics for same‐day, co‐located evaluation.

Finally, unlike the ACS/GSV program, COGS is not a verification framework or policy‐based standard. Rather, it is an operational, patient‐level intervention focused on real‐time clinical assessment and care coordination. While some ACS/GSV–aligned programs rely primarily on multidisciplinary case review and targeted specialty referrals without consistent in‐person geriatric evaluation, COGS emphasizes direct patient assessment within surgical clinics, coupled with immediate interdisciplinary communication and implementation of tailored care pathways.

In summary, while COGS aligns with the shared goals of POSH, VA, and ACS efforts, it is distinct in its embedded structure, real‐time surgeon–geriatrician collaboration, and pragmatic implementation strategy. It complements existing models by offering a scalable approach that operationalizes perioperative geriatric principles within routine surgical practice, particularly in settings where traditional geriatric‐led clinic models may not be feasible.

## 2 Program Description

Development of the COGS program followed principles of the Quality Enhancement Research Initiative (QUERI) Alignment, Commitment, Tailoring, Informing the Field, Observing Healthcare Changes, and Generating New Questions framework [27].

Alignment was achieved by ensuring the program supported Veterans Health Administration (VHA) priorities for high‐risk populations and perioperative quality improvement. Commitment was secured through dedicated institutional support, including 1.0 full‐time equivalent (FTE) geriatrician and 1.0 FTE registered nurse (RN) coordinator embedded within surgical clinics.

The program was tailored to local needs across seven surgical clinics. Veterans were screened using the Risk Analysis Index–Clinical (RAI‐C) through pre‐clinic RN review, same‐day screening, or direct geriatric assessment. The RAI‐C tool categorizes patients as robust (≤ 29), normal (30–36), frail (37–44), or very frail (≥ 45) [28]. Although a score ≥ 37 (frail or very frail) prompted comprehensive evaluation, Veterans were also evaluated at the request of surgical teams regardless of RAI‐C score.

**FIGURE 1:**
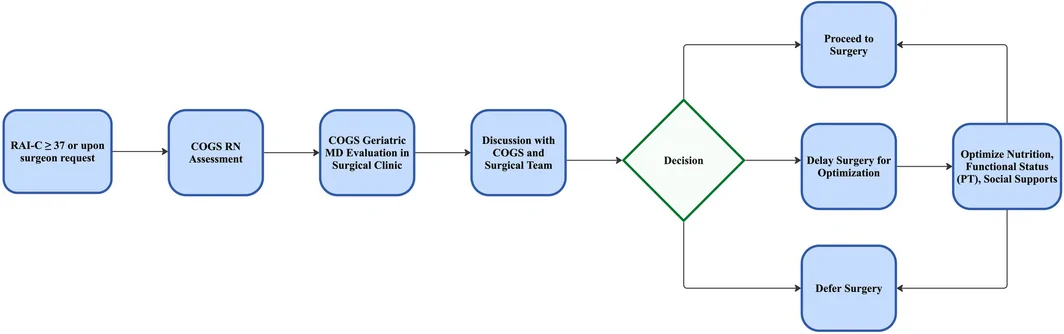
Care Coordination and Optimization in Geriatric Surgery (COGS) workflow. Older adult Veterans, aged 65 or older, with RAI‐C scores ≥ 37 or referred by surgeons undergo RN assessment and geriatrician evaluation in the surgical clinic. Findings are reviewed with the surgical team to guide decisions to proceed with surgery, delay surgery for optimization, or defer surgery.

The embedded geriatrics team conducted structured preoperative assessments aligned with the 4Ms framework of Age‐Friendly Health Systems, including review of comorbidities, medications, functional status, cognition, and social determinants of health [29, 30]. Risk estimation incorporated VASQIP [28], NSQIP [31], CAN score [32], E‐Prognosis [28], RAI‐C [28], and 6CIT [33] assessments. Goals‐of‐care discussions emphasized “What Matters Most,” and recommendations to address delirium prevention, mobility optimization, nutrition, polypharmacy, and postoperative support planning. Refer to Figure 1 for COGS program workflow.

Clinic volume averaged approximately six new consults per week, with 1–3 consults per surgical clinic session. Follow‐up was typically conducted by phone.

For collection of data, we used a privacy secured shared Excel document for data collection which included baseline characteristics of participants, RAI‐C scores, surgery types, decisions on whether to proceed with surgery, mortality and other outcomes measures. Refer to Table 1 for details. This structured data collection allowed for evaluation of program effectiveness and supported ongoing quality improvement.

**TABLE 1:** COGS attributes based on frailty score.

Characteristic	Total^a^	Robust	Normal	Frail	Very frail
Sample size	295	38	44	109	104
RAI‐C score	Range 0–81	Range 0–29	Range 30–36	Range 37–44	Range 45–81
Age	78.1 ± 6.6	73.8 ± 6.6	76.2 ± 5.4	78.3 ± 6.1	80.3 ± 6.7
Male	286 (97%)	36 (13%)	42 (15%)	107 (37%)	101 (35%)
Race					
White, non‐Hispanic^b^	272 (92%)	36 (13%)	40 (15%)	103 (38%)	93 (34%)
Non‐White	23 (8%)	2 (9%)	4 (17%)	6 (26%)	11 (48%)
Home life					
Rural^c^	59 (20%)	5 (8%)	12 (20%)	19 (32%)	23 (39%)
ADL score^d^	4.8 ± 1.34	5.2 ± 0.72	5.3 ± 0.96	5.0 ± 1.11	4.1 ± 1.69
IADL score^e^	4.7 ± 1.62	5.5 ± 0.83	5.3 ± 1.46	4.9 ± 1.28	3.9 ± 1.95
Living alone	46 (16%)	10 (22%)	12 (26%)	12 (26%)	12 (26%)
No identifiable support	51 (17%)	8 (16%)	12 (24%)	15 (29%)	16 (31%)
Surgical specialty					
General	54 (18%)	1 (2%)	9 (17%)	21 (39%)	23 (43%)
Vascular	72 (24%)	4 (6%)	12 (17%)	30 (42%)	26 (36%)
Orthopedic	78 (26%)	24 (31%)	15 (19%)	26 (33%)	13 (17%)
Urologic	67 (23%)	4 (6%)	5 (7%)	20 (30%)	38 (57%)
Consults					
PT/Prehab	45 (15%)	12 (27%)	7 (16%)	16 (36%)	10 (22%)
Nutrition	13 (4%)	1 (8%)	0	7 (54%)	5 (38%)
Nutrition Rx/no consult	87 (29%)	12 (14%)	12 (14%)	37 (43%)	26 (30%)
Goals of care					
What matters most discussed	211 (72%)	24 (11%)	33 (16%)	80 (38%)	74 (35%)
Advance directives					
New/updated directive	49 (17%)	11 (22%)	7 (14%)	18 (37%)	13 (27%)
First surrogate identified	63 (21%)	15 (24%)	7 (11%)	23 (37%)	18 (29%)
New DNR decision	37 (13%)	2 (5%)	4 (11%)	15 (41%)	16 (43%)
Incorrect surrogate	17 (6%)	2 (12%)	4 (24%)	9 (53%)	2 (12%)
First non‐NOK surrogate	17 (6%)	2 (12%)	1 (6%)	12 (71%)	2 (12%)
First LST note	119 (40%)	24 (20%)	19 (16%)	40 (34%)	36 (30%)
Consult findings					
New dementia diagnosis	17 (6%)	3 (18%)	4 (24%)	3 (18%)	7 (31%)
New impaired decision‐making	31 (11%)	3 (10%)	4 (13%)	6 (19%)	18 (58%)
Surgery versus no surgery					
Had Surgery	155 (53%)	30 (19%)	27 (17%)	56 (36%)	42 (27%)
No Surgery	140 (47%)	8 (6%)	17 (12%)	53 (38%)	62 (44%)
Reasons for no surgery					
Mutual: never	46 (16%)	1 (2%)	3 (7%)	18 (39%)	24 (52%)
Mutual: not now	53 (18%)	2 (4%)	8 (15%)	23 (43%)	20 (38%)
Veteran decline	5 (2%)	1 (20%)	2 (40%)	1 (20%)	1 (20%)
Surgeon decline	7 (2%)	1 (14%)	2 (29%)	1 (14%)	3 (43%)
Not indicated	24 (8%)	2 (8%)	2 (8%)	7 (29%)	13 (54%)
Other	5 (2%)	1 (20%)	0	3 (60%)	1 (20%)
Observed deaths					
Deaths ≤ 30 days surgery	1 (0%)	0	0	0	1 (100%)
Deaths ≤ 180 days surgery	7 (2%)	0	0	3 (43%)	4 (57%)
30‐day deaths post‐consult	4 (1%)	1 (25%)	0	1 (25%)	2 (50%)
180‐day deaths post‐consult	31 (11%)	1 (3%)	2 (6%)	7 (23%)	21 (68%)

Abbreviations: ADL, activities of daily living; DNR, do not resuscitate; IADLs – instrumental activities of daily living; LST, life sustaining treatment; NOK, next of kin.

^a^ Total column percentages calculated using column totals; frailty subcategory percentages calculated using row totals.

^b^ Missing data for 12 individuals; data was corrected and updated via chart review.

^c^ Missing data for 33 individuals, imputed as non‐rural.

^d^ Missing data for 30 individuals; mean and standard deviation were calculated without missing data.

^e^ Missing data for 32 individuals; mean and standard deviation were calculated without missing data.

## 3 Preliminary Data

Between May 30, 2023, and December 31, 2024, the COGS program conducted over 300 consults. Participant characteristics stratified by frailty category are summarized in Table 1.

**FIGURE 2:**
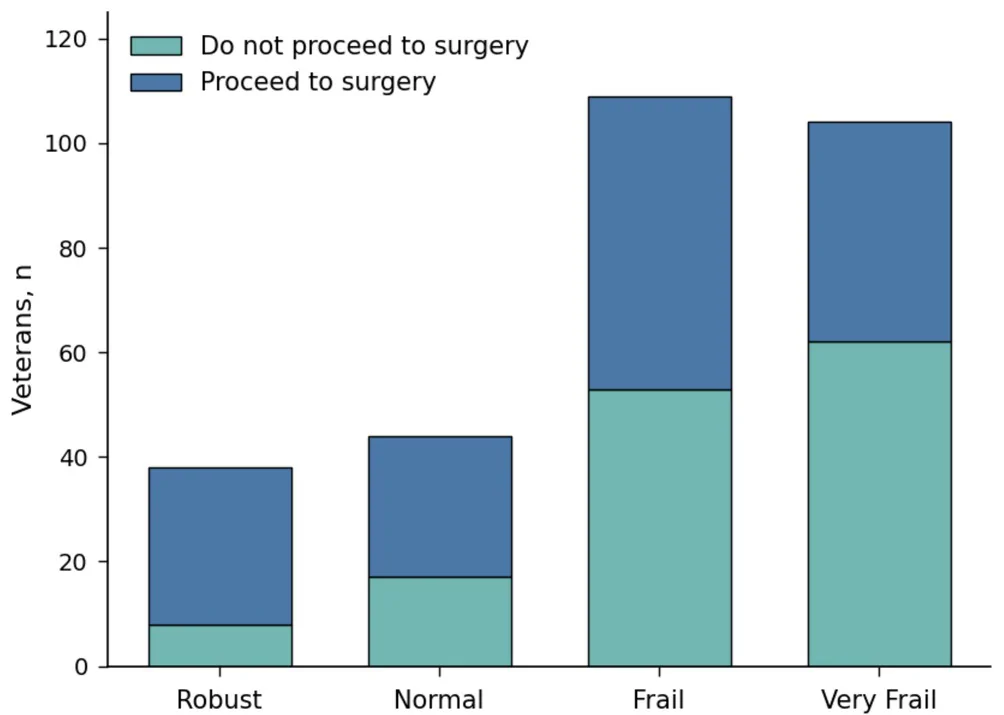
Surgical decision outcomes by frailty category. Stacked bar chart demonstrating the number of Veterans who proceeded to surgery versus those who did not, stratified by Risk Analysis Index–Clinical (RAI‐C) frailty category. RAI‐C score of ≤ 29 was characterized as robust, RAI‐C score of 30–36 was normal, RAI‐C score of 37–44 was frail, and RAI‐C score of ≥ 45 was very frail. A greater proportion of Veterans classified as frail and very frail did not proceed to surgery compared with robust and normal groups. This data suggest a clear trend that as RAI‐C score increases for a patient, so do the odds that the patient will not undergo surgery.

Overall, 155 Veterans (53%) proceeded to surgery and 140 (47%) did not. As shown in Table 1 and visually represented by Figure 2, Veterans categorized as frail or very frail were more likely not to proceed with surgery compared to robust or normal groups. For example, among very frail Veterans, 44% did not undergo surgery, compared to 21% of the robust group.

Thirty‐one deaths occurred within 180 days of COGS consultation (11%). Of these, seven occurred within 180 days following surgery. Most deaths occurred among Veterans classified as frail or very frail. These mortality data are presented descriptively. In the absence of a comparator group, no causal inference can be made regarding the effect of the COGS intervention on mortality outcomes.

Missing data included race for 12 individuals which were filled in via chart review, patient rurality (*n* = 33) imputed as non‐rural, and for ADL (*n* = 30) and IADL (*n* = 32) data in which the mean and standard deviation were calculated without missing data. These most likely resulted from incomplete capture during the COGS RN assessment and geriatric provider evaluation.

In terms of patient satisfaction, preliminary surveys indicate that Veterans have expressed high levels of satisfaction with the COGS program, appreciating the comprehensive, individualized care they received. The program has also been well‐received by surgical colleagues, who recognize the value of having geriatricians embedded in surgical clinics to optimize patient outcomes.

## 4 Discussion

A core element of the COGS model is clarifying general goals of care and patient‐specific goals for surgery, ensuring they align with likely surgical outcomes. Traditional preoperative assessments followed an informed consent model focused on estimated surgical risks and benefits, with decisions framed primarily in statistical terms. Surgeons are now encouraged to adopt the “Best Case, Worst Case, and Most Likely Case” framework, which expands these discussions to include functional recovery, quality of life, and the potential need for rehabilitation or skilled care [34, 35, 36]. Geriatricians complement this informed consent process by focusing on “What Matters Most” to the patient—clarifying surgical goals, confirming understanding of outcomes, and facilitating follow‐up discussions with the surgeon when needed [15, 28]. As part of the COGS evaluation, geriatric and surgical teams collaborate to review risks, benefits, optimization strategies, and referrals.

In contrast to centralized geriatric preoperative clinics such as POSH or standards‐based frameworks such as the ACS/GSV program, the COGS model emphasizes co‐location and same‐day interdisciplinary evaluation. Narrative reviews of collaborative medical–surgical models describe a spectrum of co‐management approaches, ranging from referral‐based assessments to fully embedded interdisciplinary care structures, reinforcing the conceptual foundation for models such as COGS [37]. This approach may enhance communication efficiency and streamline risk clarification at the point of surgical decision‐making.

Importantly, the mortality data reported here are observational and descriptive. Without a comparator group, it is not possible to determine whether outcomes differ from standard care or from other perioperative models. These findings should therefore be interpreted as hypothesis‐generating.

Implementation of the COGS program required overcoming several operational challenges.

Early barriers included limited dedicated clinic space, which necessitated flexible use of shared surgical clinic rooms and close coordination with administrative staff to align COGS visits with existing surgical appointments. Scheduling constraints were addressed by embedding consultation slots within high‐volume clinic sessions and prioritizing co‐location with surgical services to minimize patient travel and fragmentation of care. Workforce demands were substantial, particularly given reliance on a 1.0 FTE geriatrician and 1.0 FTE registered nurse; workflow standardization, templated assessments, and structured phone‐based follow‐up were implemented to maximize efficiency. Securing institutional buy‐in required ongoing engagement with surgical leadership, presentation of early process metrics, and alignment of COGS goals with system priorities such as quality improvement and perioperative risk reduction. Given finite capacity and the broader national shortage of geriatricians, we intentionally prioritized consultation for Veterans at highest risk—based on frailty, multimorbidity, and surgical complexity—while remaining responsive to surgeon‐initiated referrals. This targeted approach allowed the program to deliver high‐value, goal‐aligned perioperative care within existing resource constraints while building the foundation for future expansion.

## 5 Conclusions and Future Directions

The COGS program demonstrates the feasibility of embedding geriatric expertise directly within surgical clinics to support frailty‐informed decision‐making and coordinated perioperative care for older Veterans. While early findings describe patterns of surgical decision‐making and mortality among a high‐risk cohort, further comparative and longitudinal evaluation is needed to determine impact on clinical outcomes. This embedded model offers a structured framework for integrating geriatrics principles into surgical care within the VHA and potentially in other health systems.

Scalability of the COGS model is feasible with thoughtful workforce adaptation, particularly in the context of the well‐documented national shortage of geriatricians. Across 7 surgical clinics, the program is currently operating within a large tertiary hospital setting and receiving referrals sufficient to support 1–3 new patient evaluations per day, with a substantial proportion of referred patients able to be seen in‐person, including many on the same day as their surgical visit when scheduling allowed. Expansion of this model to provide coverage across all surgical clinics at our institution would require an estimated 3.0 FTE total (1.5 FTE for a geriatrician and 1.5 FTE nurse), recognizing that certain lower‐risk services (e.g., ophthalmology) may require only as‐needed consultation rather than dedicated session coverage. While the present model relies on a dedicated geriatrician, expansion could incorporate geriatric‐trained advanced practice providers to extend consult capacity and improve efficiency. Additionally, structured telehealth follow‐up offers an opportunity to enhance reach across geographically dispersed clinics, optimize continuity, and reduce in‐person burden while maintaining geriatric expertise within perioperative care pathways.

Building on our initial successes, we plan to expand the COGS program across all surgical clinics at our institution and develop an inpatient pathway to support high‐risk Veterans. We also aim to extend follow‐up into the post‐discharge period in coordination with the Salt Lake City VA's transition of care team. These plans are contingent on further funding.

An additional future direction of the COGS program is to expand systematic data collection on both patient‐centered and clinical outcomes. These include functional recovery, quality of life, patient satisfaction, goal‐concordant care, and surgical morbidity and mortality. In addition, the program aims to evaluate its potential to reduce postoperative delirium and lower readmissions and healthcare costs. Formal reporting of these findings will be essential to inform program refinement, demonstrate value, and support dissemination.

## Author Contributions

**Gabrielle A. Scannell:** acquisition of subjects and data, interpretation of data, writing and reviewing of manuscript. **Carole A. Baraldi:** acquisition of subjects and data, original programmatic concept and design, writing and reviewing of manuscript. **Randall Rupper:** original programmatic concept and design, data collection decisions, reviewing of manuscript. **Michelle T. Mueller:** original programmatic concept and design, reviewing manuscript. **Rachel J. Brenner:** acquisition of subjects and data, writing and reviewing of manuscript. **Thomas White:** analysis and interpretation of data, and review of manuscript. **Brian C. Sauer:** analysis and interpretation of data, and review of manuscript. **Jared Lareno Hansen:** analysis and interpretation of data, and review of manuscript. **G. Paul Eleazer:** original programmatic concept and design, data collection decisions, development, writing and review of manuscript.

## Funding

This work was funded by the U.S. Department of Veterans Affairs (VA), Office of Research and Development (ORD). This material is the result of work supported with resources and the use of facilities at the VA Salt Lake City Health Care System. The contents do not represent the views of the Department of Veterans Affairs or the United States government.

## Disclosure

This content in this paper has not been submitted or presented at a meeting. It also has not been published previously. Startup funding for this project was provided by the Veterans Integrated Service Network (VISN) 19, and subsequent funding was provided by the Salt Lake City Veterans Health Care System (SLC VHCS). Neither VISN 19 nor SLC VHCS was involved in the design, methods, subject recruitment, data collection, analysis nor the preparation of this manuscript.

## Consent

Written consent has been obtained from everyone named in the Acknowledgments section.

## Conflicts of Interest

The authors declare no conflicts of interest.
